# Pulmonary manifestation of a condition resembling Kasabach–Merritt syndrome in a woman with abdominal angiomatosis associated with consumptive coagulopathy – surgical management: a case report

**DOI:** 10.1186/s13256-015-0566-z

**Published:** 2015-05-09

**Authors:** Danjouma Housmanou Cheufou, Thomas Hager, Stefan Welter, Dirk Theegarten, Georgios Stamatis

**Affiliations:** 1 Department of Thoracic Surgery, Ruhrlandklinik-Essen, University of Duisburg-Essen, Essen, Germany; 2 Institute of Pathology and Neuropathology, University Hospital of Essen, University of Duisburg-Essen, Essen, Germany

**Keywords:** Abdominal angiomatosis, Hemoptysis, Kasabach–Merritt syndrome, KMS

## Abstract

**Introduction:**

Kasabach–Merritt syndrome is a benign condition characterized by hemangiomatosis, severely disseminated intravascular consumption coagulopathy, and thrombocytopenia. The mortality rate increases from 12% to 30% in hemorrhagic cases. In general, the symptoms primarily manifest in the gastrointestinal tract, the skin, and the subcutaneous tissue. There is no publication about pulmonary manifestation of angiomatosis in combination with vascular malformation and hemoptysis. This is the first description of a Kasabach–Merritt syndrome-like condition in the lung.

**Case presentation:**

We present the case of a 29-year-old German woman with angiomatosis and associated pulmonary vascular malformation in her lower left lobe with a Kasabach–Merritt syndrome like condition. It was detected after hemoptysis. We also present our case observations and management.

**Conclusion:**

In a case of angiomatosis with an associated pulmonary lobar vascular malformation, lobectomy can be safely performed to prevent life-threatening bleeding.

## Introduction

Angiomatosis is defined as a diffuse proliferation of well-developed blood vessels. It is a benign condition, continuously affecting a large segment of the body. Formerly classified as neoplasm, it is currently defined as vascular malformation. Approximately two-thirds of the cases manifest in the first 2 decades of life [1]. Kasabach–Merritt Syndrome (KMS) was first described in 1940 in a newborn male patient who presented thrombocytopenic purpura associated with rapidly growing capillary hemangioma [2-4]. Since then, the term KMS has been used to describe different cases which widely match the initial description [2]. In fact, the term “hemangioma”, used in the literature for describing a range of vascular anomalies, leads to difficulties in distinguishing “true KMS” from entities with similar clinical presentation [2]. This, in turn, causes confusion concerning clinical presentations and outcomes of patients with “true KMS” [3,4].

The etiology of the KMS remains unclear. It has been hypothesized that exposure of the subendothelial elements or abnormal endothelium within the hemangioma results in aggregation and activation of platelets with a secondary consumption of clotting factors [2,3,5,6]. The primary treatment includes stopping the consumption coagulopathy, inducing regression of the angioma, and resecting the hemangioma while preventing major bleeding [7,8]. Several therapy regimes have been proposed to achieve this aim. Here we present a case which was classified as KMS and treated with lower left lobectomy.

## Case presentation

**Figure 1:**
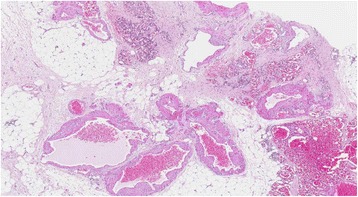
Histological examination of the initial abdominal “tumor”. Fat tissue with different vascular patterns ranging from small capillary and cavernous vessels to thick-walled vessels with broadened intima (hematoxylin and eosin staining).

**Figure 2:**
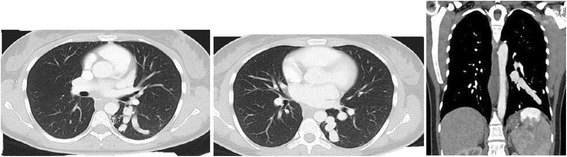
A chest computed tomography scan showing varicose veins of the lower lobe.

**Figure 3:**
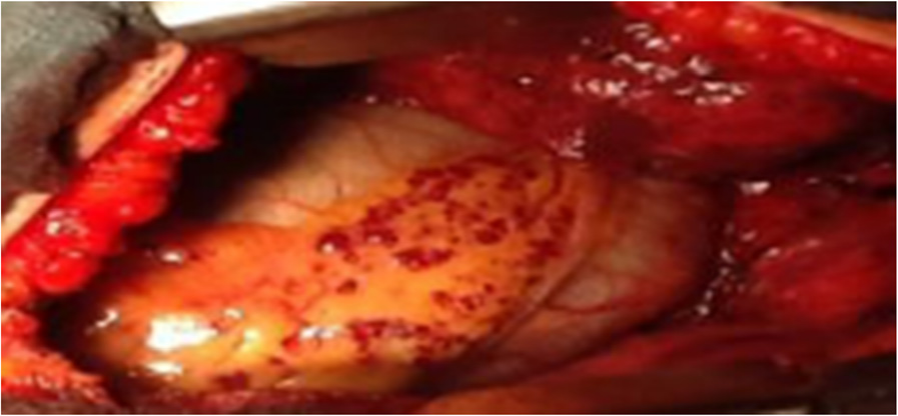
Intraoperative view on the blood clots in the varicose veins of the paracardial fat.

**Figure 4:**
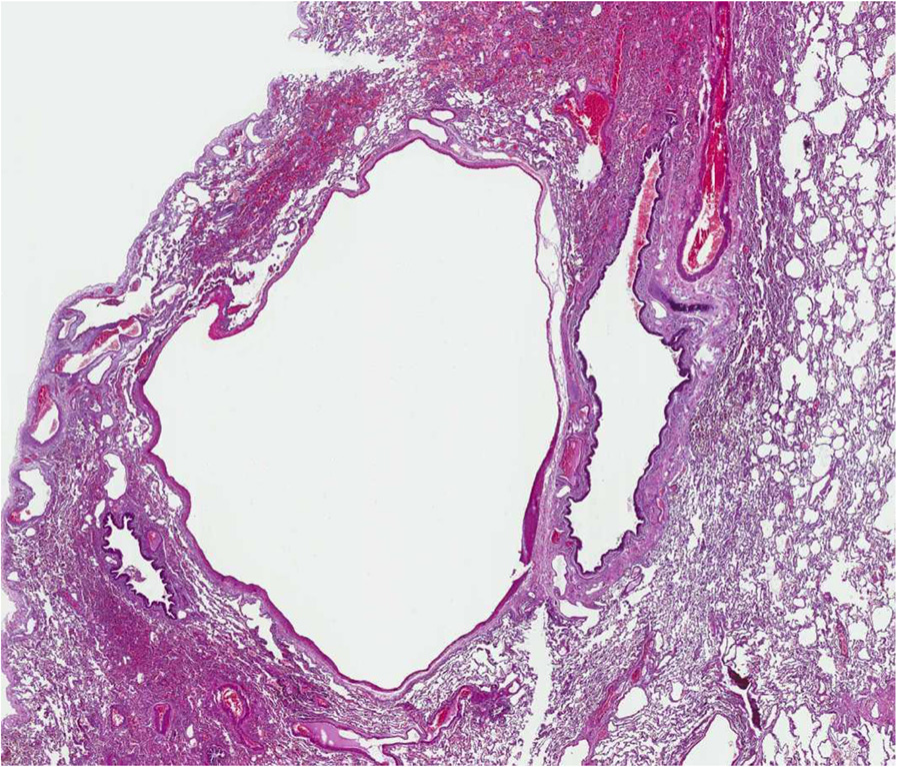
Histological examination of the resected lower left lung lobe. Lung specimen with solitary vascular malformation and residual hemorrhages in the neighboring parenchyma (hematoxylin and eosin staining).

A 29-year-old German woman was admitted to our hospital with a history of hemoptysis a few days before admission. She did not report severe coughing, emesis, or extraordinary physical stress. She had been admitted to another hospital 15 years ago because of significant gastrointestinal bleeding caused by esophageal varices of 2nd to 3rd degree. A preoperative computed tomography (CT) scan showed an upper abdominal mass with a potential infiltration of her liver. Furthermore, a thrombosis of her portal vein and a partial thrombosis of her superior mesenteric vein were detected. She displayed a splenomegaly with a strong lienal vein and a paravertebral vascular plexus. She therefore underwent laparotomy. During surgery, the lesion was diagnosed as a hemangiomatosis of the greater omentum, extending through the hepatoduodenal ligament to the left lobe of her liver; varicose veins of her entire stomach wall were also detected. Because of this extension, the lesion was classified as non-resectable. A biopsy (Figure 1) of the tumor was performed, and an end-to-end portacaval anastomosis was created. The biopsy was examined by a consultant pediatric pathologist (Kiel, Germany) and categorized as an angiomatosis. Her postoperative course was uneventful. Both a KMS and an eventual hereditary telangiectasia (Osler–Weber–Rendu syndrome) were considered. Finally, because of the clinical presentation, the disease pattern was classified as “KMS”. Corticosteroid therapy was initiated for 3 months, followed by interferon alpha (IFN -α) for an additional 3 months. The patient survived the following 15 years without symptoms and then returned with hemoptysis. On admission, she was clinically asymptomatic and hemodynamically stable. Her platelet number was 139,000/μL (150,000 to 450,000/μL) and a gastroscopy revealed grade I esophageal varices without signs of bleeding. The walls of her esophagus, stomach, and upper small intestine were swollen. A chest radiograph revealed a shadow on the lower left lobe of her lung. Thereafter, a CT scan of her thorax and abdomen was performed. We observed a vasodilation in the lower left lobe of her lung and, in particular, a dilation of her intrapulmonary veins (Figure 2). The upper left lobe and her right lung were not affected. After interdisciplinary discussion of this case, we decided to perform a lower left lobectomy to treat hemoptysis and prevent life-threatening bleeding. We performed an open lower left lobectomy through a muscle-sparing antero-axillary thoracotomy. The motivation to choose an open procedure and not a thoracoscopic intervention was to avoid involuntary lung manipulation and allow control of the vulnerable dilated vessels (Figure 3). A histopathological examination of the resected specimen showed solitary focal vascular malformations with associated fresh and old residual hemorrhages of the neighboring tissue (Figure 4). These features may reflect “KMS”. Her postoperative course was uneventful. She is still alive 2 years after the lobectomy without any clinical symptoms of KMS.

## Discussion

The presented case reflects the condition of an abdominal angiomatosis with associated solitary vascular malformation of the lung and KMS-like characteristics. The most frequent sites involved with angiomatosis are the lower extremities; followed by the chest wall, the abdomen and upper extremities [1]. Most reported cases of KMS displayed hemangiomas involving the skin, in severe cases visceral sites and the retroperitoneum [2]. In this case, the radiological findings of the chest CT scan showed a major dilatation of the intrapulmonary vein originating from the lower left lobe; therefore, we chose resection of the lower left lobe to avoid major bleeding in the course of hemoptysis.

Numerous therapeutic approaches are available for the treatment of KMS. Various nonsurgical therapies have been reported for the treatment of hemangioma, such as corticosteroids, IFN-α, vincristine, and radiotherapy (RT); however, their use is limited due to their side effects [9]. One of the proposed regimens is the administration of corticosteroids. These drugs inhibit angiogenesis, which is secondary to a variety of effects including enhanced sensitivity of the vascular bed and the circulation of endogenous vasoconstrictive agents [10]. Other response patterns include a partial decrease of the lesion size or an improvement of the bleeding disorder with little effect on the hemangioma. In the treatment of KMS, glucocorticosteroids yield a heterogeneous response, ranging from no effect to regression of the hemangioma, and moderate to complete suppression of coagulopathy [11].

Corticosteroids are often used in combination with RT, IFN-α, or surgery. IFN-α inhibits angiogenesis by suppressing overexpression of the basic fibroblast growth factor, an angiogenic protein, in infantile hemangiomas. Fost and Esterly [12] treated 24 pediatric patients with hemangioma using IFN-α; they documented a complete response in 42%, a substantial response in 16%, an intermediate response in 26%, and no response in 16% of the patients. However, many patients develop neurological deficits caused by IFN-α treatment, especially when initiated at an early age. These symptoms may disappear after the treatment is discontinued.

In the past, RT was the treatment of choice for hemangiomas and KMS. Approximately 85% of the cases of hemangioma treated with RT prior to 1960 showed complete resolution, whereas 15% showed considerable improvement. On occasion, hemangiomas are resistant to RT [5]. Several late effects of RT have been evaluated and reported, such as malignancies and growth retardation. These effects are closely related to the total dose and volume of the treatment and to the employed techniques. When RT is chosen, its lowest dose should be used to decrease the occurrence of possible late effects and secondary malignancies. However, this also causes a decrease in the probability of successful therapy. Consequently, none of the nonsurgical approaches were chosen for the presented case because no compartment could spatially restrict the bleeding. In a case of a lobar manifestation of KMS, lobectomy can be safely performed to prevent life-threatening bleeding.

## Conclusion

In a case of angiomatosis with an associated pulmonary lobar vascular malformation, lobectomy can be a promising option to prevent life-threatening bleeding.

## Consent

Written informed consent was obtained from the patient for publication of this case report and any accompanying images. A copy of the written consent is available for review by the Editor-in-Chief of this journal.

## Abbreviations

CT: Computed tomography
IFN-α: Interferon alpha
KMS: Kasabach–Merritt syndrome
RT: Radiotherapy
